# Endogenous target mimics down-regulate miR160 mediation of *ARF10*, *-16*, and *-17* cleavage during somatic embryogenesis in *Dimocarpus longan* Lour

**DOI:** 10.3389/fpls.2015.00956

**Published:** 2015-11-05

**Authors:** Yuling Lin, Zhongxiong Lai, Qilin Tian, Lixia Lin, Ruilian Lai, Manman Yang, Dongmin Zhang, Yukun Chen, Zihao Zhang

**Affiliations:** Institute of Horticultural Biotechnology, Fujian Agriculture and Forestry UniversityFuzhou, China

**Keywords:** *Dimocarpus longan*, somatic embryogenesis, microRNA160, endogenous target mimics, auxin response factors, gene regulation

## Abstract

MicroRNA160 plays a critical role in plant development by negatively regulating the auxin response factors *ARF10*, *-16*, and *-17*. However, the ways in which miR160 expression is regulated at the transcriptional level, and how miR160 interacts with its targets during plant embryo development, remain unknown. Here, we studied the regulatory relationships among endogenous target mimics (eTMs), and miR160 and its targets, and their involvement in hormone signaling and somatic embryogenesis (SE) in *Dimocarpus longan*. We identified miR160 family members and isolated the miR160 precursor, primary transcript, and promoter. The promoter contained *cis*-acting elements responsive to stimuli such as light, abscisic acid, salicylic acid (SA) and heat stress. The pri-miR160 was down-regulated in response to SA but up-regulated by gibberellic acid, ethylene, and methyl jasmonate treatment, suggesting that pri-miR160 was associated with hormone transduction. Dlo-miR160a, -a^∗^ and -d^∗^ reached expression peaks in torpedo-shaped embryos, globular embryos and cotyledonary embryos, respectively, but were barely detectable in friable-embryogenic callus. This suggests that they have expression-related and functional diversity, especially during the middle and later developmental stages of SE. Four potential eTMs for miR160 were identified. Two of them, glucan endo-1,3-beta- glucosidase-like protein 2-like and calpain-type cysteine protease DEK1, were confirmed to control the corresponding dlo-miR160a^∗^ expression level. This suggests that they may function to abolish the binding between dlo-miR160a^∗^ and its targets. These two eTMs also participated in 2,4-D and ABA signal transduction. *DlARF10*, *-16*, and *-17* targeting by dlo-miR160a was confirmed; their expression levels were higher in friable-embryogenic callus and incomplete compact pro-embryogenic cultures and responded to 2,4-D, suggesting they may play a major role in the early stages of longan SE dependent on 2,4-D. The eTMs, miR160, and *ARF10*, *-16*, and *-17* exhibited tissue specificity in ‘Sijimi’ longan vegetative and reproductive organs, but were not significant negatively correlated. These results provide insights into the possible role of the eTM-miR160-*ARF10-16-17* pathway in longan somatic embryo development.

## Background

MicroRNAs, a conserved class of single-stranded non-coding RNAs of approximately 21 nt in length, are derived from pri-miRNAs containing imperfect stem-loop secondary structures (Jones-Rhoades et al., 2006). These hairpin RNAs, which are known as pre-miRNAs genes, are transcribed by RNA polymerase II in plants, processed by DICER- LIKE1 to produce an miRNA:miRNA^∗^ duplex in the nucleus, and transported to the cytoplasm (Lee et al., 2004; Jones-Rhoades et al., 2006). The mature miRNA recognizes target mRNA sites through perfect or near perfect complementarity, and negatively regulates mRNA expression by cleaving targeted mRNAs (Llave et al., 2002) or repressing translation (Gandikota et al., 2007). miRNAs play vital roles in a range of developmental processes (Carrington and Ambros, 2003; Jones-Rhoades et al., 2006). The expression of miRNA genes is tightly controlled. Recently, a novel mechanism involving eTMs was discovered, whereby miRNA–mRNA duplexes with a large bulge around the cleavage site actually sequester miRNAs, resulting in inhibition of miRNA activity (Franco-Zorrilla et al., 2007). eTMs have been used as a valuable tool to understand the functions of several miRNA families in plants (Wu et al., 2013).

A highly conserved miRNA family, miR160, negatively regulates *ARF10* (Liu et al., 2007), *-16* (Wang et al., 2005), and *-17* (Mallory et al., 2005) of the repressor ARF family, and plays a critical role in maintaining the process of seed germination and the normal developmental programs of embryos, roots, stems, leaves, siliques, and floral organs. An *Arabidopsis* loss-of-function mutation in miR160a caused various embryonic defects, suggesting that miR160 may control embryo development (Liu et al., 2010b). Ectopic expression of miR160 results in auxin hypersensitivity, cytokinin hyposensitivity, and inhibition of symbiotic nodule development in soybean (Turner et al., 2013), and decreases seed dormancy in *Arabidopsis* (Liu et al., 2013). The over-expression of artificial target mimics for miR160 resulted in plants of a smaller size with serrated and upward-curled leaves (Todesco et al., 2010; Yan et al., 2012). Over-expression of eTMs of miR160 also caused smaller and serrated leaves in transgenic plants (Wu et al., 2013), suggesting that eTMs play a key role in plant development by inhibiting miRNA activity. However, to date, analysis and identification of eTMs for miR160 has rarely been performed in plants (Wu et al., 2013; Shuai et al., 2014).

*Dimocarpus longan* Lour., also called longan or dragon eye, is a tropical tree of the soapberry family (Sapindaceae) that produces edible fruit. As a traditional medicine, longan fruit is used to enhance memory (Park et al., 2010), promote blood metabolism, relieve insomnia, and prevent amnesia (Yi et al., 2011). Longan seed extracts, which contain high levels of antioxidant polyphenolic compounds (Chung et al., 2010) and polysaccharides (Zheng et al., 2010), are capable of inducing apoptosis in various colorectal cancer cells. Previous studies have shown that longan’s embryo development status influences its seed size, fruit quality, fruit set and yield (Liu et al., 2010a; Lai and Lin, 2013; Lin and Lai, 2013a,b). However, because of the extreme genetic heterozygosity exhibited by this species and the difficulty of sampling early embryos, advances in understanding of the regulation of longan embryo development have been limited.

Somatic embryogenesis, which resembles zygotic embryogenesis, is an essential component of plant cell differentiation and embryo development. The longan SE system has been used widely as a model system to investigate *in vitro* and *in vivo* regulation of embryogenesis in plants (Lai et al., 2010; Lin and Lai, 2010). In our previous study, we found that miR160a was barely detectable during the early stages of longan SE, but highly expressed during the heart- and torpedo-shaped embryonic stages (Lin and Lai, 2013b). Previous reports have suggested that larch and *Brassica napus* miR160s might play a regulatory role during cotyledonary embryo development (Zhang et al., 2012; Huang et al., 2013). However, it is not clear how post-transcriptional regulation of *ARF10*, *-16*, and *-17* by miR160 regulates plant embryo development or SE, particularly in longan.

The aim of this study was to investigate the regulatory networks of eTMs and miR160-mediated regulation of *ARF10*, *-16*, and *-17* in longan, especially during SE. Here, we identified and examined the expression of mature miR160 molecules, the miR160 primary transcript and its promoter, eTMs, and homologs of the target genes *ARF10*, *-16*, and *-17* for miR160. Our results suggest possible general regulation by eTM down-regulation of miR160 cleavage of *ARF10*, *-16*, and *-17* during SE in longan.

## Materials and Methods

### Tissue Samples

Synchronized embryogenic cultures at different developmental stages from *D. longan* ‘Honghezi’ (Fujian, China), consisting of EC, ICpEC, GE, HE, TE, and CE, were generated as detailed in Lai and Chen (1997).

The EC was pre-cultured on Murashige and Skoog (MS) medium containing 2,4-D (1.0 mg/L) for 20 days, and then sub-cultured in 50 mL of liquid MS medium containing 2,4-D (0, 0.5, 1.5, or 2 mg/L), SA (75 μM), MeJA (50 μM), ET (30 ppm), GA_3_ (35 μM), or ABA (100 μM) in a rotary shaker at 150 rpm for 24 h. These cultures were all kept in the dark.

*Dimocarpus longan* ‘Sijimi’ samples, including roots, leaves, floral buds, flowers, young fruits, mature fruits, pericarp, pulp and seeds, were collected from the experimental fields of Fujian Academy of Agricultural Science in Putian. All materials from at least six rootstock plants of the same cultivar were combined.

The synchronized embryogenic cultures, EC cultures treated with different hormones, and ‘Sijimi’ samples were collected and stored at -80°C for subsequent analyses.

### Database Searching for miR160 Members of Longan

Previously published small RNA data (BioSample accession SAMN04120614, Bio-Project ID PRJNA297248) (Lin and Lai, 2013b), generated using various synchronized embryogenic cultures from the longan ‘Honghezi’ variety, was used to identify miR160 family members. The potential miR160 members were searched using the miRBase server (Release 21: June 2014). Multiple alignment analysis of miR160 members was performed using DNAMAN ver. 6.0 (Lynnon Biosoft Corp, Pointe-Claire, QC, Canada).

### Pri-miR160 3′ and 5′ RACE Experiments

To determine whether the miR160 members were present in the *D. longan* genome, the full-length cDNA sequence of pri-miR160 was cloned by RT-PCR and 3′/5′ RACE PCR from the EC and mixed cDNA. The EC cDNA for RT-PCR of the pre-miR160 conserved regions was synthesized using a RevertAid First Strand cDNA Synthesis Kit (Fermentas, Thermo Fisher Scientific, Waltham, MA, USA). The mixed cDNA for pri-miR160 3′/5′ RACE PCR was synthesized from mixed total RNA (EC, ICpEC, GE and HE) using a GeneRacer^TM^ Kit (Invitrogen, Corporation, Carlsbad, CA, USA), following the manufacturer’s instructions.

The amplification of the full-length cDNA of pri-miR160 was carried out by 3′/5′ RACE PCR from the longan mixed cDNA, and its genomic sequence was cloned from the EC DNA template. The sequences of all primers used are provided in **Table 1**.

**Table 1:** Gene names and primer sequences.

Gene name	Primer sequence (5′-3′)	Description
primiR160-F	TGCCTGGCTCCCTGTATGC	Precursor cloning
primiR160-R	ATGCATGGCTCCTCATACGC	
primiR160-F	TGCCTGGCTCCCTGTATGC	*3*′*RACE*
primiR160-F1	GCGTACGAGGAGCCATGCA	
primiR160-R	ATGCATGGCTCCTCATACGC	*5*′*RACE*
primiR160-R1	GCATACAGGGAGCCAGGCA	
primiR160-CF	CGCGACTCTACCATATTCACTAA	Full length confirmation
primiR160-CR	CCCCGAAACAAACTTCGTTCTT	
160-SP1	CCATCTACTACACAGCATGCATTCG	Promoter cloning
160-SP2	CCTTCCATGTTGGTGCGGTGACAT	
160-SP3	GGCATTCTGGAGCTATATAGCCATAAC	
160-SP4	GATATAGTGATGAGGGTAGCAGGTTC	
160-SP5	GATATAGTGATGAGGGTAGCAGGTTC	
160-SP6	GCTATAACGAACTCATGTTGGCATAC	
ARF1-5RACE1	GAAGTCTGTGTAGAATGCGCCATG	miR160 cleavage site identification
ARF10-5RACE2	CTGGATGATTGGACTAGTGGAG	
ARF16-5RACE1	GAGTGGGATTTCCCATCGTAAGC	
ARF16-5RACE2	GGAGTAGGATGATGATGATGCTG	
ARF17-5RACE1	CTACTTAACCAAATGTCATACGCAACG	
ARF17-5RACE2	CTGTTGAGCAGTGCCGTGTAAGTC	

To determine the phylogeny of the miR160 gene, we downloaded all precursor sequences of plant miR160s from miRBase release 21. Phylogenetic analysis was performed according to the neighbor-joining method (Saitou and Nei, 1987) using MEGA 5.02 (Kumar et al., 2004), with 1000 bootstrap replicates.

### miR160 Promoter Cloning and *cis*-Acting Element Analysis

To study the molecular mechanism underlying the transcriptional regulation of miR160, genome walking was performed using TAIL-PCR (Takara, Japan) to obtain the miR160 promoter. The promoter was cloned by three nested PCR amplifications from the longan EC DNA template with the same forward primer AP2 and specific reverse primers. The primer sequences can be found in **Table 1**. *Cis*-acting element analysis of the miR160 promoter was carried out using the PlantCARE database (Lescot et al., 2002).

### Prediction of eTMs for the miR160 Family in *D. longan*

First, the longan EC transcriptome database (SRA050205) (Lai and Lin, 2013) was used to search for sites in the cDNA sequences that were reverse complements of the miR160 family, and the tool TAPIR was used to search for eTMs according to criteria described previously (Bonnet et al., 2010). The cDNAs that satisfied the above criteria were considered to be target mimic candidates.

### Modified 5′ RLM-RACE

To determine the internal cleavage sites in the *ARF10*, *-16*, and *-17* mRNAs, a modified 5′ RLM-RACE experiment was performed using a GeneRacer kit (Invitrogen). Briefly, total RNAs from equal mixtures of EC, GE, HE, and TE were ligated to a 5′ RACE RNA adapter. The experiment essentially followed the manufacturer’s instructions. The PCR amplification used GeneRacer 5′ primers and gene-specific primers (**Table 1**). The resulting PCR products were gel-purified, cloned, and sequenced.

### Gene Expression Assays

Total RNAs were isolated from the nine synchronized embryogenic cultures described above using the TRIzol Reagent kit (Invitrogen). cDNAs for miRNA and mRNA quantification were synthesized using a One Step PrimeScript^®^ miRNA cDNA Synthesis Kit (Takara Code, D350A), and PrimeScript^TM^ Perfect Real Time RT Reagent Kit (TaKaRa Code, DR037A), respectively.

Quantitative PCR assays of gene expression were performed using a LightCycler 480 qPCR instrument (Roche Applied Science, Switzerland) and SYBR premix (Takara). All reactions were performed in triplicate. The expression levels of the miR160s, their targets (*ARF10*, *-16*, *-17*), and the eTMs were quantified using internal standards (Lin and Lai, 2010, 2013c). Statistical analysis was performed using SPSS 19. Gene names and primer sequences are provided in **Table 2**.

**Table 2:** Gene names and primer sequences for qPCR analysis.

Gene name	Primer sequence (5′-3′)
dlo-miR160a-F	CTGGCTCCCTGTATGCCA
dlo-miR160a^∗^-F	CGTATGAGGAGCCATGCATA
dlo-miR160d^∗^-F	GCGAGGAGCCATGCAT
PremiR160-qF	GTAGATGGTATGCCTGGCTCC
PremiR160-qR	TAGCGAGCATGCATGGCT
64827F(77)	CCATCCTTGTCGTGGCTA
64827R(256)	TGGGACTACTGGAATCAATGA
66891F(193)	AGAACCTGATGGACTCCCAG
ARF10-QF	TTCCTGGCAACCTCCTTG
ARF10-QR	CGAAAACAGACCTGACTGCA
ARF16-QF	CCTGTCATGATCAGCAGCC
ARF16-QR	CGACTGCAGCTTGTTGAAGT
ARF17-QF	CGAGGACATCTTCTCTCCACTG
ARF17-QR	CACGTCTGATGAGTATTCTCGC

## Results

### Analysis of the miR160 Family Members from a Longan Small RNA Dataset

**FIGURE 1:**
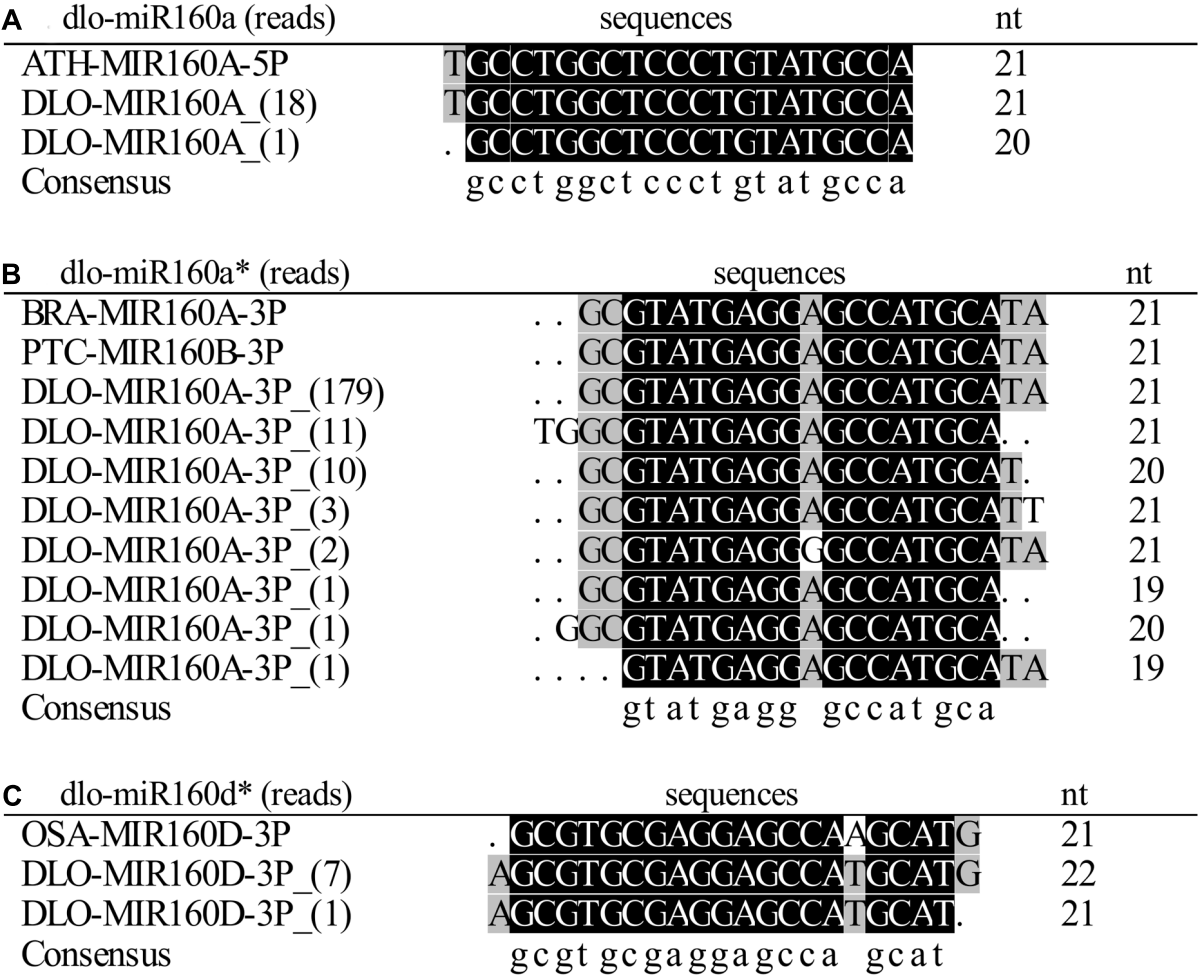
**Alignment of *Dimocarpus longan* miR160 members. (A)** miR160a; **(B)** miR160a^∗^; **(C)** miR160d^∗^. ATH, *Arabidopsis thaliana* (miRBase No. MI0000190); BRA, Brassica rapa (miRBase No. MIMAT0010155); PTC, *Populus trichocarpa* (miRBase No. MIMAT 0022890); OSA, *Oryza sativa* (miRBase No. MIMAT0022854); DLO, *Dimocarpus longan.*

The miR160 family, comprising three members (ath-miR160a–c) in *Arabidopsis* and six members (osa-miR160a–f) in *Oryza sativa*, was first identified in plants (Reinhart et al., 2002). By searching a longan small RNA dataset, we found that the miR160 family was represented by 12 members with different read counts (**Figures 1A–C**). The 12 potential miR160 members were then compared against the miRBase database (Release 21), which revealed that they were distributed into two isoforms. One was identical to ath-miR160a, including ten similar variants; the other was identical to osa-miR160d, including two variants. We named these two isoforms dlo-miR160a and dlo-miR160d, respectively. However, variants similar to miR160b and miR160c were not found. Therefore, there are at least two miR160 isoforms in the *D. longan* genome. According to the Blast alignment results, of these 12 variants, only two mature miRNAs were excised from the 5′ arm of the hairpin, the others were located in the 3′ arm of the hairpin.

### Identification of the miR160 Gene Sequence in Longan

**FIGURE 2:**
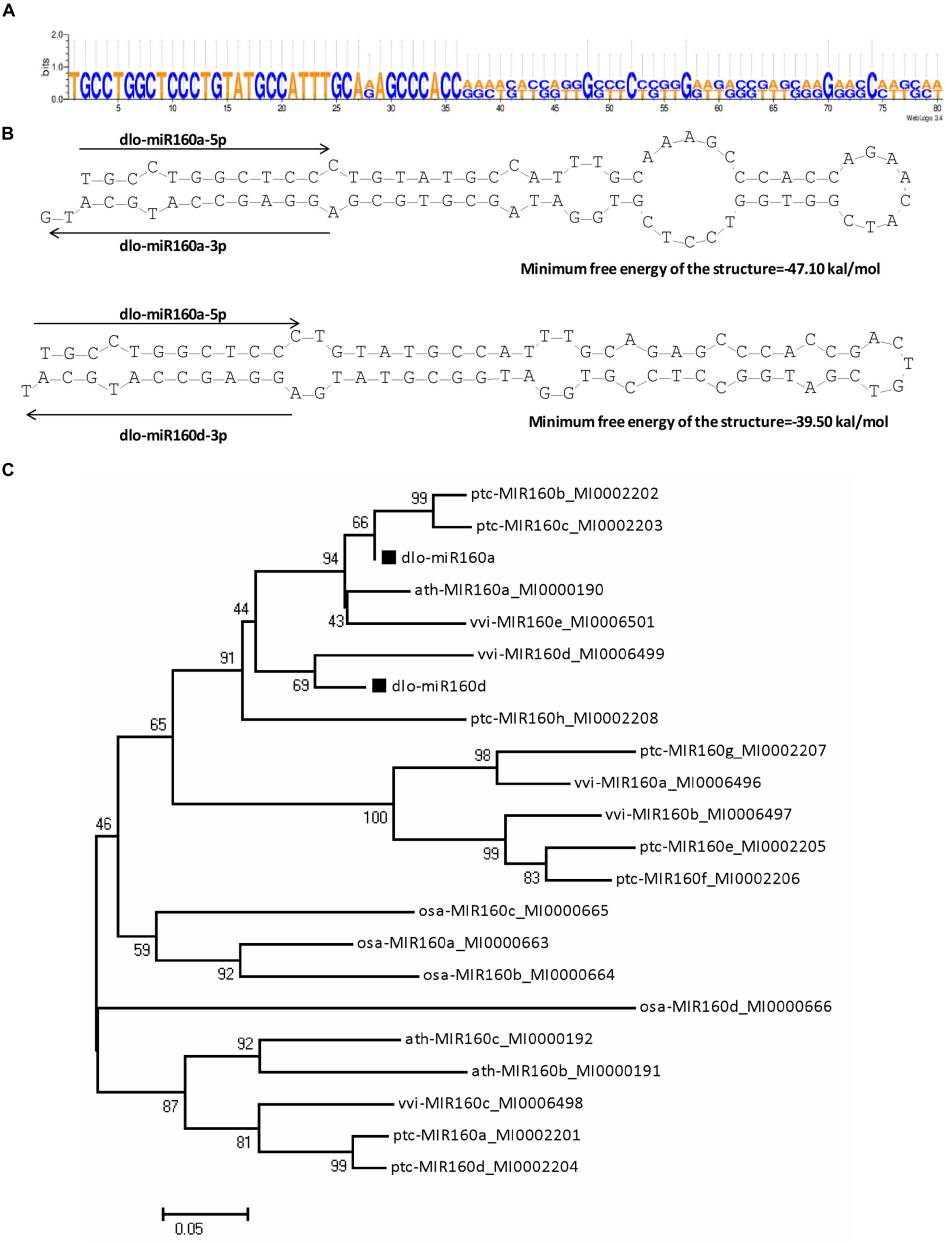
**Separate primary MIR160 transcripts contain dlo-miR160a/a^∗^ and -160d^∗^. (A)** Longan miR160 precursors. **(B)** Hairpin structures of the longan miR160 precursors containing dlo-miR160a/a^∗^ and -160d^∗^. **(C)** Neighbor-joining phylogenetic tree of pre-miR160 sequences in plants. Numbers in the tree represent bootstrap values. ptc, *Populus trichocarpa*; vvi, *Vitis vinifera*; dlo, *Dimocarpus longan;* ath, *Arabidopsis thaliana*; osa, *Oryza sativa*.

To find the miR160 gene and determine its structure and length in the *D. longan* genome, the full-length cDNA sequence of pri-miR160 was obtained by 3′/5′-RACE. Two precursors with lengths of 80 and 82 bp were obtained by RT-PCR. Alignment analysis showed that these two precursors were highly homologous except for 13 nucleotide differences (**Figure 2A**). To determine the hairpin structure of the miR160 precursors and the positions of the miR160 members, secondary structure analysis of the precursors was conducted using the DNAMan6.0 software. The two miR160 precursors both formed the classic stem-loop structure, and minimum free energy (MFE) analysis indicated that the stabilities of their secondary structures were -47.1 and -39.5 kcal/mole, respectively (**Figure 2B**). By comparing these two precursors and the mature miR160 sequences, dlo-miR160a-5p variants were found that exactly matched the 5′ arms of both precursors, while dlo-miR160a-3p and -d-3p variants were detected in the 3′ arms of the two precursors, respectively. The sequence that exactly matched the dlo-miR160a-3p variants was named pre-miR160a; the other one was identical to dlo-miR160d-3p and was named pre-miR160d (**Figure 2B**). Phylogenetic analysis (**Figure 2C**) also suggested that the pre-miR160a sequence in *D. longan* was homologous to pre-miR160 sequences in *Arabidopsis* (miR160a), *Populus trichocarpa* (miR160b, c), and *Vitis vinifera* (miR160e). Pre-miR160d appeared to be closely related to *V. vinifera* miR160d.

Next, 5′ and 3′ RACE analyses for the pri-miR160 transcripts were carried out. The full-length sequence was 441 bp (GenBank accession No. KJ372214), with the typical poly(A) tail in the 3′ UTR, and the first nucleotide (transcription initiation site) of the full-length cDNA was A. BLAST analysis of the pri-miR160 showed similarities with existing pri/pre-miR160s in *Manihot esculenta*, *P. trichocarpa*, *Gossypium hirsutum*, and *Arabidopsis thaliana*. The corresponding pri-miR160 genomic DNA sequence, which comprised 424 bp and contained one 100 bp intron, was also obtained (GenBank accession No. KJ372217). The sequence at the exon–intron junctions was consistent with the eukaryotic GT–AG rule.

### Isolation of the miR160 Promoter in Longan

**FIGURE 3:**
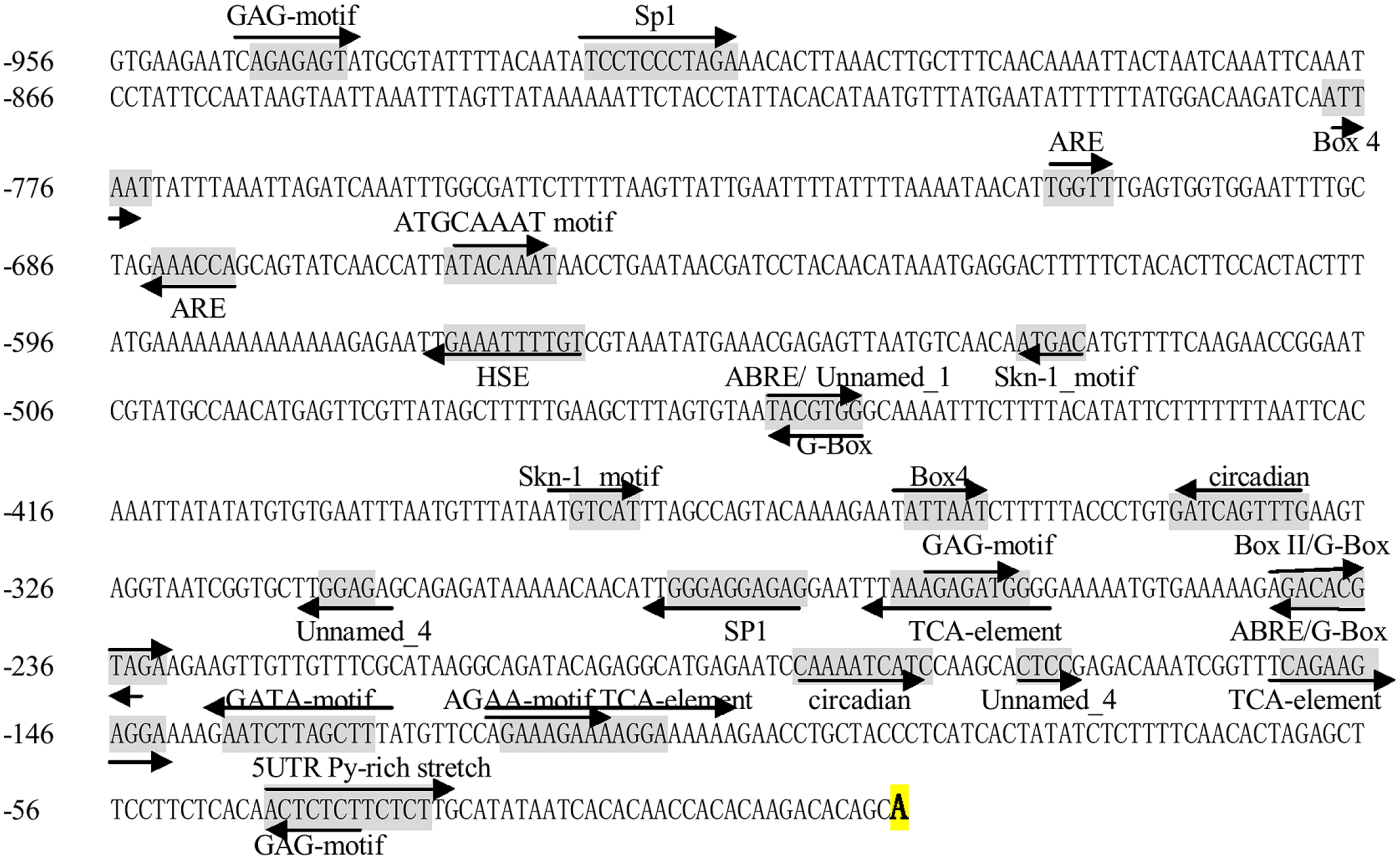
**5′ flanking sequence of pri-miR160 in *D. longan.*** The bold A is the transcription start site (TSS). *Cis*-acting regulatory elements are underlined and shaded in gray; their orientations are shown by arrows.

To further understand the regulatory role of the miR160 gene at the transcriptional level, a 957 bp 5′-flanking sequence of the pri-miR160 was cloned from longan EC (GenBank accession No. KJ372217). PlantCARE (Lescot et al., 2002) was used to search for *cis*-acting elements in the pri-miR160 promoter. The results revealed the presence of conserved TATA and CAAT boxes. Many putative *cis*-elements associated with responses to light (BOX-4, Box II, G-BOX, GAG-motif, GATA-motif and Sp1), ABA (ABRE), SA (TCA-element) and environmental stresses (heat stress-response element, HSE) were found in the promoter. In addition, an anaerobic response element (ARE) that is essential for anaerobic induction, a Skn-1_motif that is required for endosperm expression, a circadian element involved in circadian control, and four elements of unknown function were also present in the promoter (**Figure 3**). These results prompted us to carry out further experiments to identify the response of the miR160 gene to various environmental stimuli.

### Transcriptome-wide Prediction of eTMs of the miR160 Family in Longan

**FIGURE 4:**
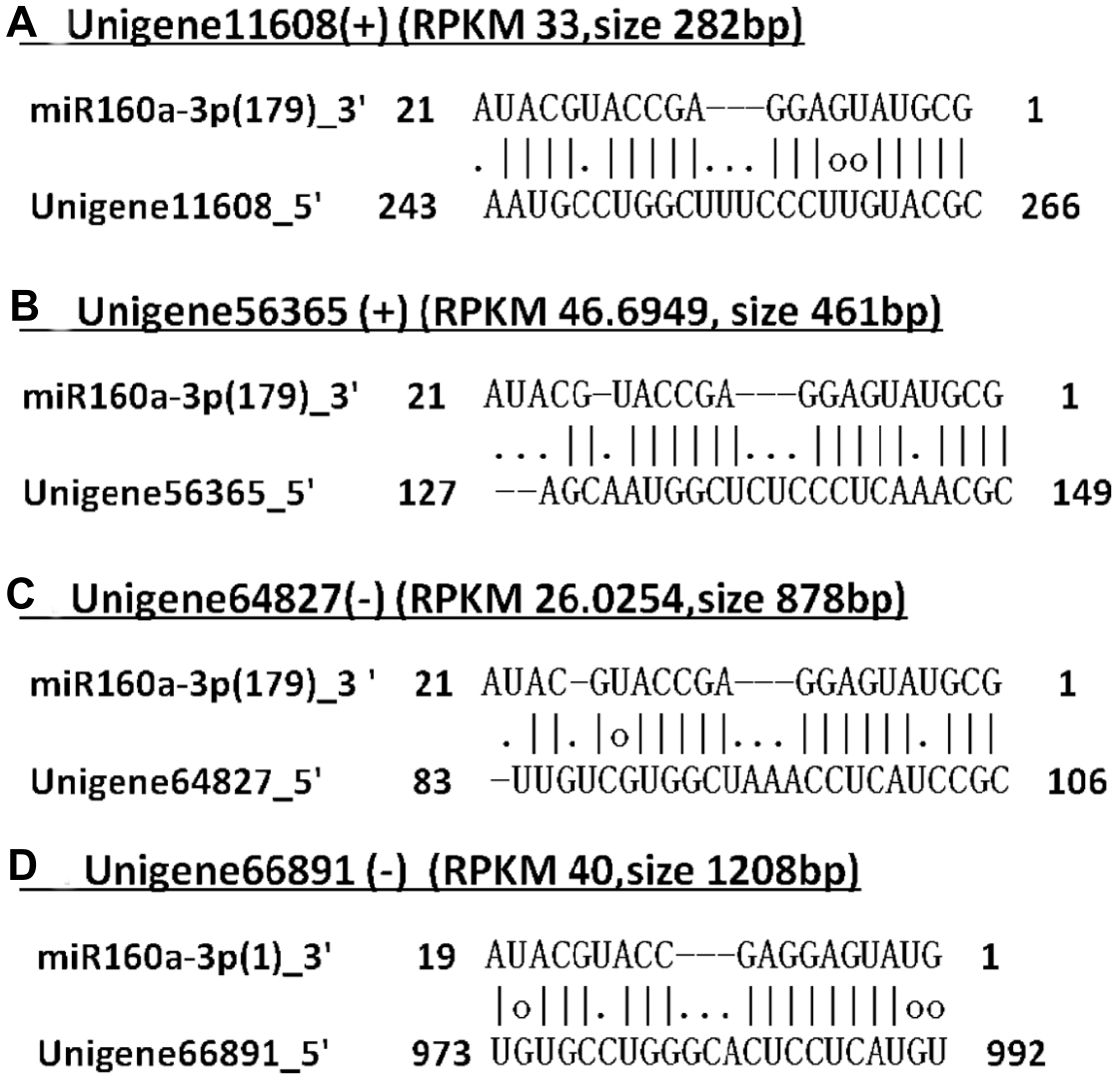
**Predicted eTMs for the miR160 family in longan.** (+), mRNA sense strand; (-), mRNA antisense strand; RPKM, reads per kilobase per million mapped reads; size, unigene length. **(A)** Unigene11608: an unknown mRNA; **(B)** Unigene56365: a nitrate transporter family protein; **(C)** Unigene64827: glucan endo-1,3-beta- glucosidase-like protein 2-like; **(D)** Unigene66891: calpain-type cysteine protease DEK1.

To date, an analysis of eTMs for miR160 has not been performed in longan. Here, transcriptome-wide computational prediction of eTMs for miR160 was performed. Four potential eTMs were identified, including an unknown mRNA (Unigene11608), a nitrate transporter family protein (Unigene56365), glucan endo-1,3-beta- glucosidase-like protein 2-like (Unigene64827), and calpain-type cysteine protease DEK1 (Unigene66891) (**Figure 4**). By contrast, two and eleven non-coding eTMs for miR160 were predicted in *Arabidopsis* and rice, respectively (Wu et al., 2013). The four potential eTMs all contained a three-nucleotide insertion between the nucleotides interacting with positions 10 and 11 of the corresponding miRNA, and the mimic sites of Unigene11608 and Unigene56365 were on the sense strand of the 3′ UTR and 5′ UTR, respectively, while the other two mimics were located on the antisense strand of the CDS (coding sequence) region (**Figure 4**). These results suggested that the miR160 family might also be controlled by eTMs in *D. longan*.

### ARF Transcripts are Cleaved by miR160 During Longan SE

**FIGURE 5:**
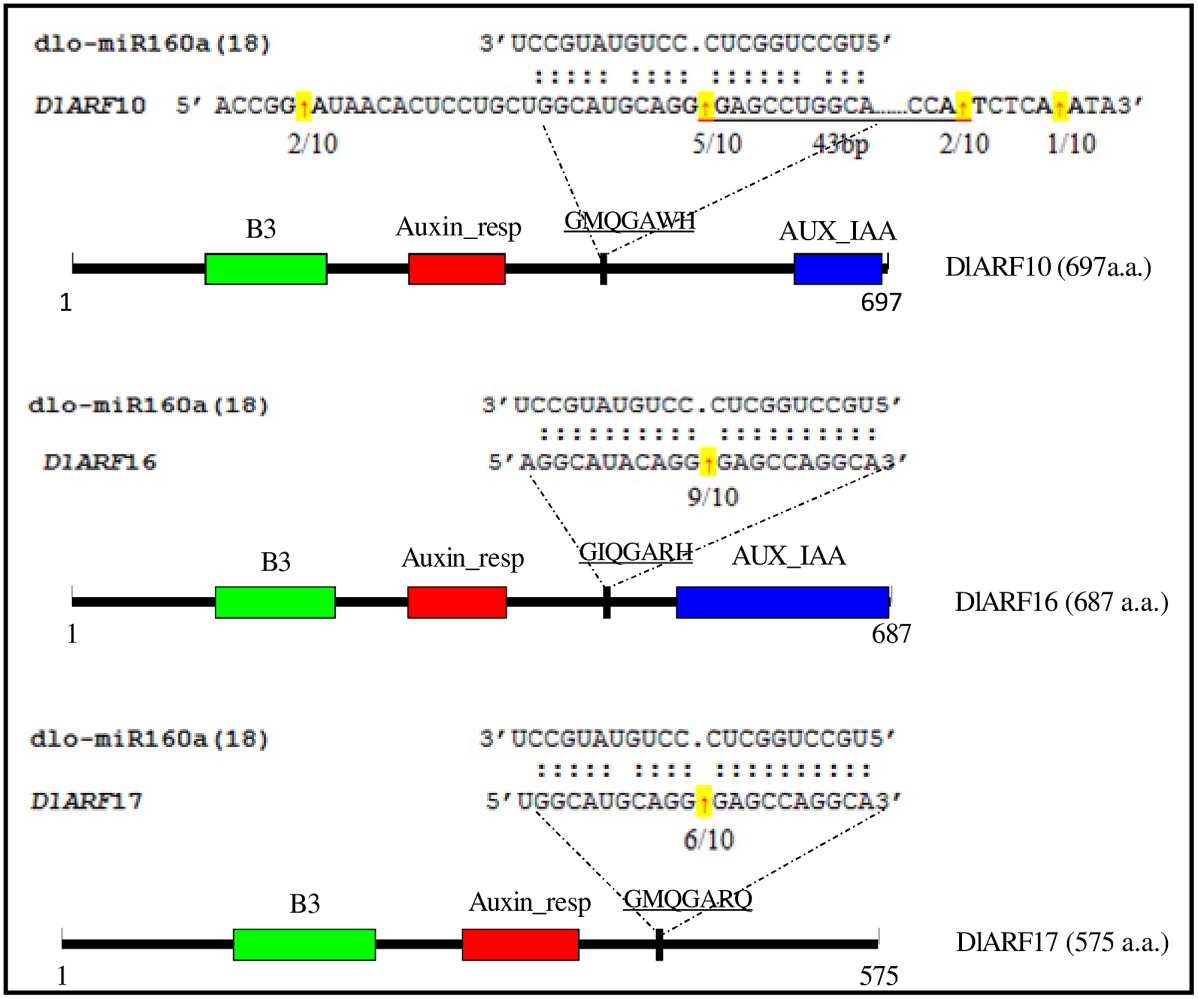
**Cleavage site mapping of dlo-miR160a target genes.** The mRNA sequences of *DlARF10*, *-16*, and *-17* are aligned with dlo-miR160a. Numbers indicate the fraction of cloned PCR products terminating at different positions. The B3 DNA binding domain (B3), Auxin_resp, and AUX_IAA are highlighted in green, red, and blue, respectively, in the ARF10, -16, and -17 proteins. The dlo-miR160a complementary sequence in the *DlARF10*, *-16*, and *-17* mRNAs and the corresponding amino acid sequences are shown.

In *Arabidopsis*, miR160 targets *ARF10*, *-16*, and *-17*, and its regulation of these transcripts appears to be important in seed germination, and shoot and root development (Mallory et al., 2005; Wang et al., 2005; Liu et al., 2007); however, its role in longan SE remains unknown. The mRNA sequences of the *D. longan* ARFs *DlARF10* (KJ410234), *-16* (KJ410235), and *-17* (KJ410237) were extracted from GenBank to search for possible miR160 cleavage sites. Alignment analysis showed that DlARF10, -16, and -17 shared high amino acid sequence similarities. All contained a conserved family domain and a B3 domain, while DlARF17 was distinguished by its poorly conserved C-terminal domain (**Figure 5**), which was consistent with the ARFs identified from *Arabidopsis* (Wang et al., 2005).

Fragments of the *DlARF10*, *-16*, and *-17* mRNAs were detected from longan embryogenic tissues and were determined to be cleaved by miR160 using a modified form of RLM-RACE. The miR160a target sites in the *DlARF10*, *-16*, and *-17* mRNAs were located at positions corresponding to the amino acid sequences GMQGAWH, GIQGARH, and GMQGARQ, respectively. The miR160a sequence showed high complementarity to these regions in *DlARF10*, *-16*, and *-17*, with only one or two mismatched bases (**Figure 5**). To determine the tissues in which miR160a regulates the *ARF10*, *-16*, and *-17* mRNAs, we searched for cleaved fragments of all three genes in longan EC cDNA and mixed cDNA samples. Fragments of the three genes were not detected in the EC samples, but were found in the mixed cDNA sample, indicating the presence of cleaved products in the mixed cDNA, but not in EC. These results clearly indicated that miR160a cleaved the *ARF10*, *-1*6, and *-17* mRNAs during longan SE, confirming that miR160-*ARF10-16-17* signal transduction operates in longan, similarly to *Arabidopsis*.

### Confirmation of an eTM-miR160-*ARF10-16-17* Regulatory Pathway During Longan SE

**FIGURE 6:**
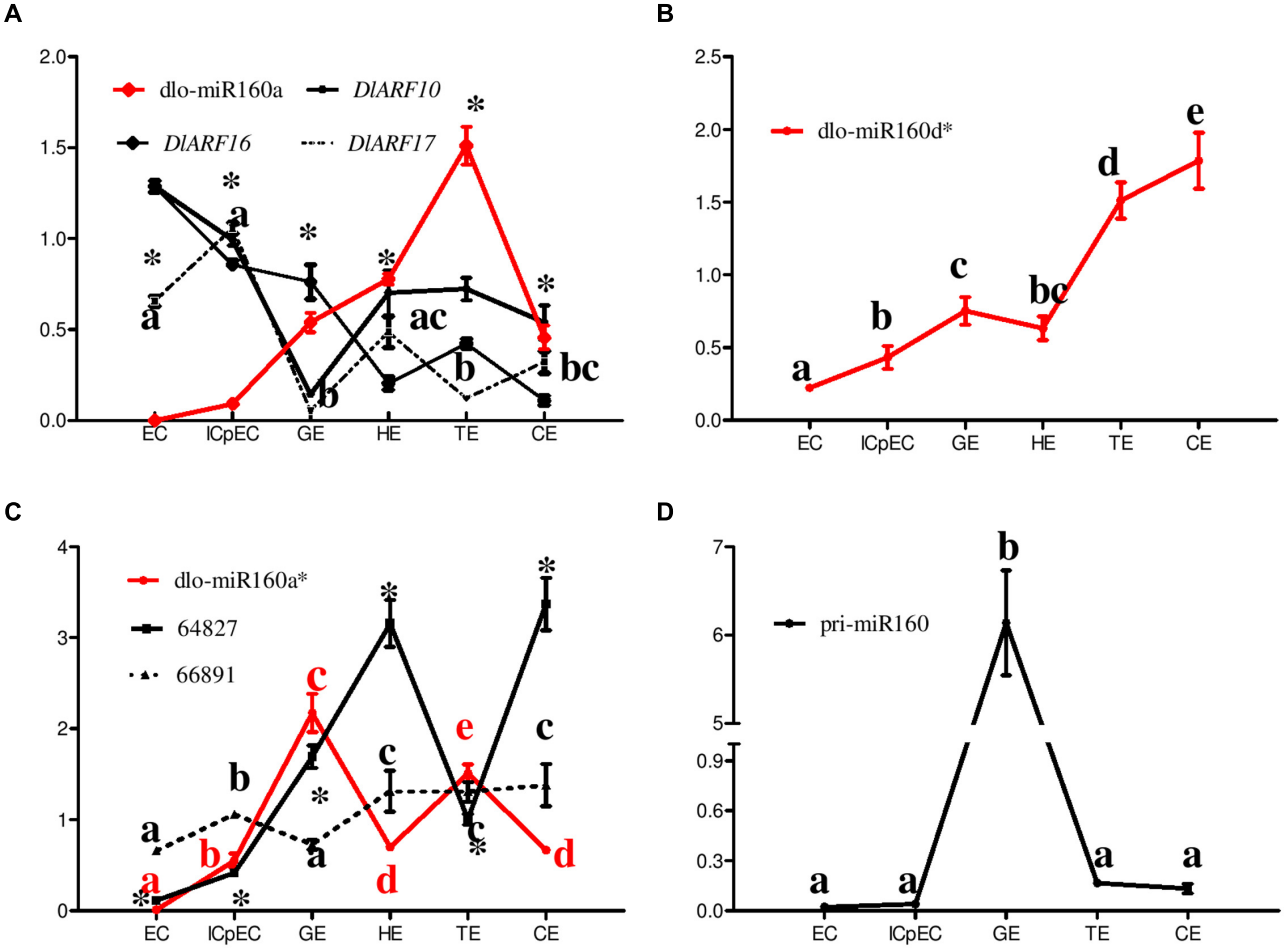
**Relative expression of eTMs (Unigene64827/66891), the dlo-miR160 family, miR160 targets and pri-miR160 during longan SE. (A–D)** The expression levels were normalized to the reference genes *DlFSD1a*, *EF-1a* and *eIF-4a*. Unigene64827: glucan endo-1,3-beta-glucosidase-like protein 2; Unigene66891: calpain-type cysteine protease DEK1 family. Samples: EC, friable-embryogenic callus; ICpEC, incomplete compact pro-embryogenic cultures; GE, globular embryos; HE, heart-shaped embryos; TE, torpedo-shaped embryos; CE, cotyledonary embryos. The y-axes represent the relative expression values; the x-axes represent longan SE samples. Asterisks (^∗^) and different lowercase letters above the bars indicate a statistically significant difference, and identical lowercase letters denote no significant difference among different embryogenic cultures (*P* < 0.05).

**FIGURE 7:**
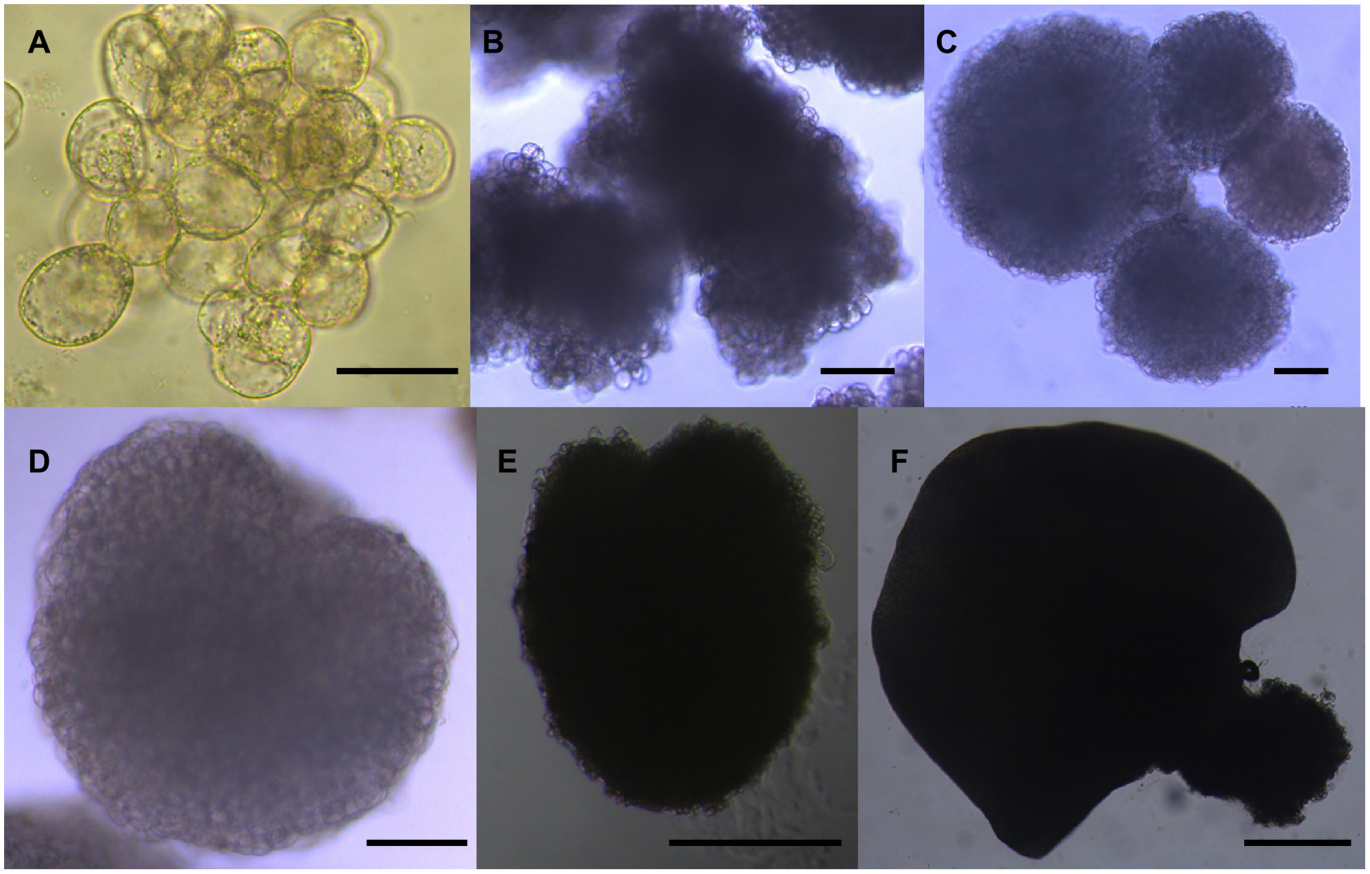
**Morphology of the six sequential developmental stages of longan SE. (A)** Friable-embryogenic callus (EC); **(B)** incomplete compact pro-embryogenic cultures (ICpEC); **(C)** globular embryos (GE); **(D)** heart-shaped embryos (HE); **(E)** torpedo-shaped embryos (TE); **(F)** cotyledonary embryos (CE). The bars are 50 μm in **(A)**, 100 μm in **(B–D)**, and 500 μm in **(E,F)**.

To determine experimentally whether the potential dlo-miR160 family members obtained from the longan small RNA data were indeed expressed, and whether the predicted eTMs function to abolish the binding between miR160 and its targets (*ARF10*, *-16*, *-17*), we investigated their expression patterns during SE in *D. longan* (**Figures 6** and **7**).

The expression levels of dlo-miR160a, -a^∗^, and -d^∗^ were investigated during *D. longan* SE (**Figures 6A–C**). Members of the dlo-miR160 family exhibited different temporal and spatial expression. Dlo-miR160a and -a^∗^ both exhibited tissue-specific expression and showed similar expression patterns; both were barely detectable in EC. The abundance of dlo-miR160a increased during the ICpEC, GE and HE stages and reached a peak in the TE stage, while dlo-miR160a^∗^ showed its highest expression in GE. In addition, dlo-miR160d^∗^ expression was lowest in EC, but continually increased in ICpEC-GE-TE, reaching its peak in the CE stage. These results showed that different members of the dlo-miR160 family might have different and separate regulatory effects on longan SE. Furthermore, the expression levels of the mature miR160s and pri-miR160 were compared. Pri-miR160 showed a similar expression pattern to dlo-miR160a^∗^, but had some differences compared with the expression levels of dlo-miR160a and -d^∗^ (**Figure 6D**).

To examine whether the four predicted eTMs for dlo-miR160a^∗^ were indeed transcribed, and to test whether these eTMs control miR160 expression, we validated their expression during longan SE. The unknown mRNA (Unigene11608) and nitrate transporter family protein (Unigene56365) were not detected in the six tissues. However, endo-1,3-beta-glucosidase-like protein 2-like (Unigene64827) and calpain- type cysteine protease DEK1 (Unigene 66891) showed great variation among the tissues (**Figure 6C**). Both were highly expressed in the HE and CE stages, and weakly expressed in EC, suggesting that they might play potential roles in the late stages of SE. Their expression patterns were largely reciprocal to that of dlo-miR160a^∗^, especially at the GE to CE stages (**Figure 6C**). These results showed that the predicted eTMs (endo-1,3-beta-glucosidase-like protein 2-like and calpain-type cysteine protease DEK1) may indeed function to abolish the binding between dlo-miR160a^∗^ and its targets.

*DlARF10*, *-16*, and *-17* targeting by dlo-miR160a members was confirmed by RLM- RACE during longan SE, and their expression levels were further examined in six tissues (**Figure 6A**). *DlARF10* and *-16* both reached their peaks in the EC stage, while *DlARF17* expression was highest in the ICpEC stage. *DlARF10* and *-17* expression was lowest in the GE stage, and *DlARF16* was expressed weakly in the CE stage. As expected, their expression was mainly inversely proportional to that of dlo-miR160a (**Figure 6**).

Taken together, these results confirmed the eTM-miR160-*ARF10-16-17* regulatory pathway might play a potential role during the SE process in *D. longan*.

### 2,4-D Controls the Levels of the eTMs, miR160, and *ARF10*, *-16*, and *-17*

**FIGURE 8:**
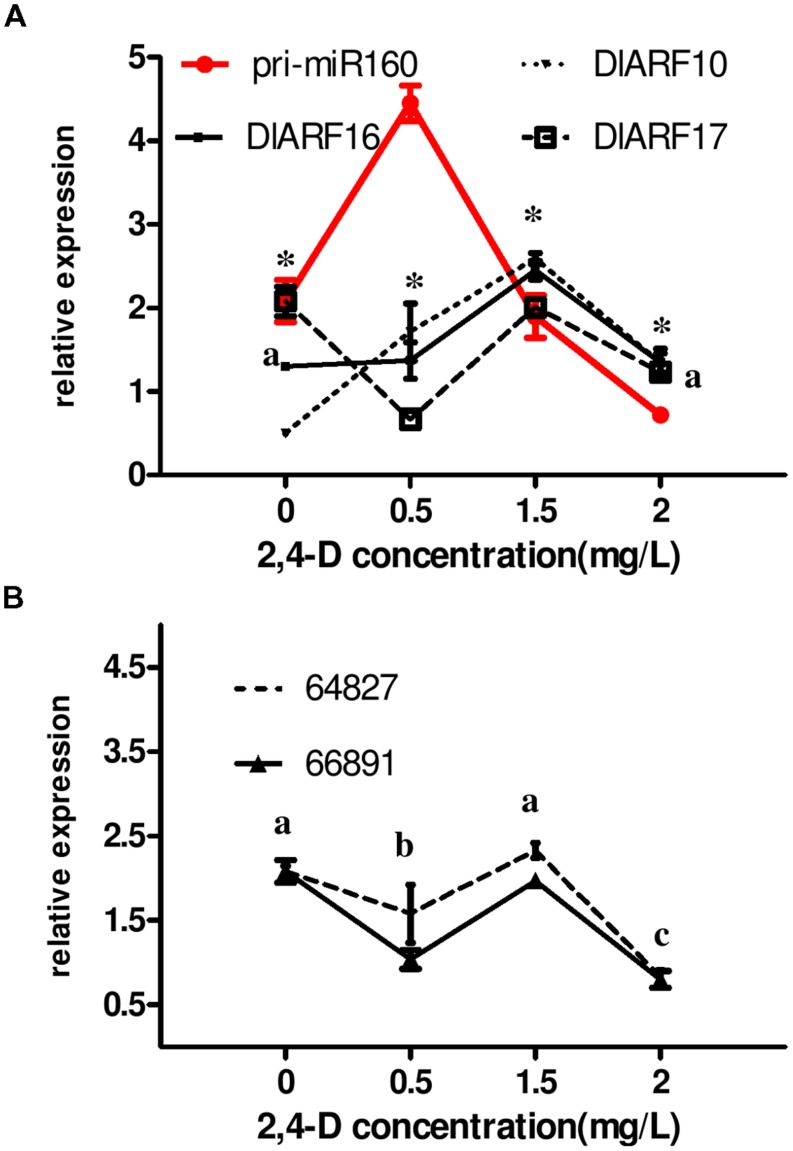
**Auxin control of eTM, miR160, and *ARF10/-16/-17* expression levels. (A,B)** The expression levels were normalized to the reference genes *DlFSD1a*, *EF-1a* and *eIF-4a*. Asterisks (^∗^) and different lowercase letters above the bars indicate a statistically significant difference, and identical lowercase letters denote no significant difference among different embryogenic cultures (*P* < 0.05).

Auxin plays major roles in embryo development. To investigate whether the miR160 level and eTM-miR160-ARF10-16-17 signal transduction were controlled by exogenous auxin, they were analyzed in longan EC grown for 24 h on media containing different concentrations of 2,4-D (an auxinic herbicide). The results showed that the 2,4-D concentration affected the eTM, miR160, and *ARF10*, *-16*, and *-17* levels (**Figure 8**).

Pri-miR160 accumulated at 0.5 mg/L 2,4-D after 24 h of treatment compared with auxin-free medium under the same conditions, but decreased significantly at higher concentrations (**Figure 8**), substantiating the participation of miR160 in the 2,4-D signal pathway. The expression patterns of the eTMs (endo-1,3-beta-glucosidase-like protein 2-like and calpain-type cysteine protease DEK1) resembled those of *DlARF16* and *-17* targeted by dlo-miR160a under the tested concentrations of 2,4-D (**Figure 8**). Both were expressed at lower levels at 0.5 mg/L 2,4-D compared with auxin-free medium, increased at 1.5 mg/L, and decreased at 2.0 mg/L, which was largely reciprocal to pri-miR160 expression. This result demonstrated that the presence of auxin in the medium affected the levels of the eTMs (endo-1,3-beta-glucosidase-like protein 2-like and calpain-type cysteine protease DEK1), and in turn, miR160- mediated regulation of *DlARF16* and *-17* in longan EC. However, the level of *DlARF10* increased as the 2,4-D concentration increased (0.5–1.5 mg/L), but decreased at 2.0 mg/L, which was not the reverse of pri-miR160. This suggested that *DlARF10* responds to 2,4-D in a positive manner, but might be out of miR160’s control.

Taken together, these results clearly demonstrated that 2,4-D controlled the levels of the eTMs (endo-1,3-beta-glucosidase-like protein 2-like and calpain-type cysteine protease DEK1), and in turn, miR160-mediated regulation of *DlARF16* and *-17* in longan. However, it is not clear whether auxin affects the eTM levels directly.

### ABA Regulates eTM-miR160 Signal Transduction

**FIGURE 9:**
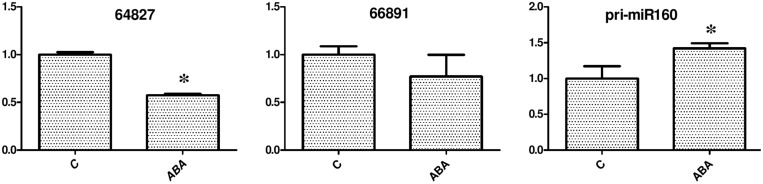
**Exogenous ABA treatment affects the eTM and miR160 expression levels.** The expression levels were normalized to the reference gene *EF-1a*. Unigene64827: glucan endo-1,3-beta-glucosidase-like protein 2, Unigene66891: calpain-type cysteine protease DEK1 family. An asterisk (^∗^) indicates a statistically significant difference between the control (C) and hormone treatments (*P* < 0.05). The *y*-axes represent relative expression values; the *x*-axes represent hormone concentration.

A previous study showed that miR160-ARF10 plays an important role in ABA-auxin crosstalk in seed germination and post-embryonic developmental programs (Liu et al., 2007). To test whether ABA treatment also affected miR160 expression and eTM-MIR160 signal transduction in longan, longan EC was grown for 24 h on medium containing 100 μM ABA and analyzed. The results showed that ABA treatment affected the eTM and miR160 levels (**Figure 9**). After ABA treatment, the levels of the eTMs (endo-1,3-beta-glucosidase-like protein 2-like and calpain-type cysteine protease DEK1) were slightly decreased compared with the control, while pri-miR160 expression was elevated after 24 h. Thus, the increased expression of pri-miR160 in longan EC after ABA treatment might be caused by decreased expression of the eTMs (endo-1,3-beta-glucosidase-like protein 2-like and calpain- type cysteine protease DEK1), or through the ABRE element in its 5′ regulatory region.

### Expression Changes of Pri-miR160 in Response to Hormone Treatments

**FIGURE 10:**
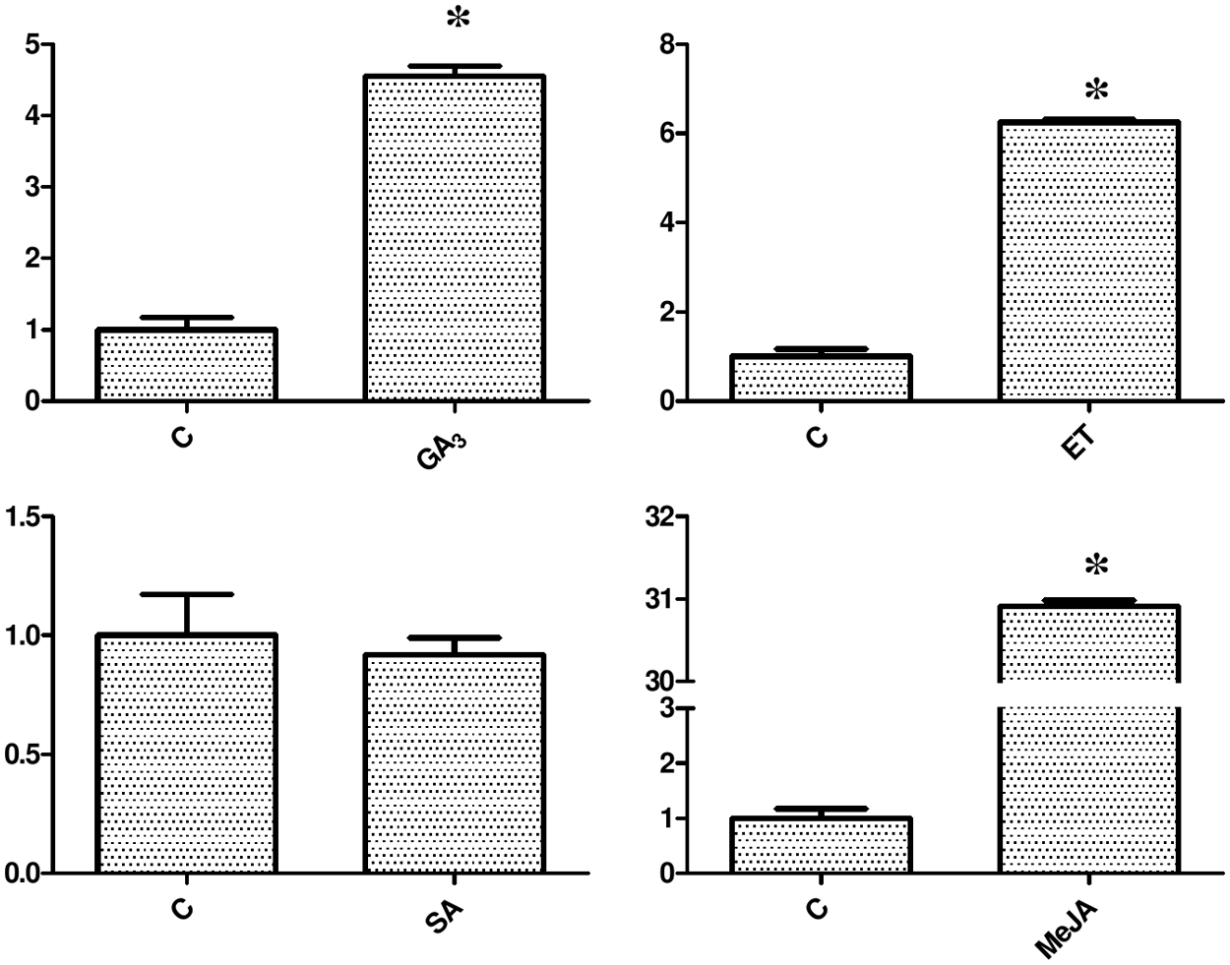
**Expression analysis of pri-miR160 in response to exogenous hormone treatments.** The expression levels were normalized to the reference gene *EF-1a*. An asterisk (^∗^) indicates a statistically significant difference between the control (C) and hormone treatments (*P* < 0.05). The *y*-axes represent relative expression values; the *x*-axes represent hormone concentration.

To further analyze the possible functions of miR160 in phytohormone signaling pathways, the expression of pri-miR160 in response to hormonal stimuli was investigated. The pri-miR160 expression level was greatly increased in response to GA_3_ (35 μM), ET (30 ppm), and MeJA (50 μM) treatments, but was slightly decreased after 24 h of SA (75 μM) treatment (**Figure 10**). Thus, GA_3_, ET, and MeJA up-regulate the expression of miR160, while SA down-regulates its expression in longan EC, possibly through related *cis*-elements in its promoter region. However, only an SA-responsive *cis*-element (TCA-element) was predicted in the promoter region. These results suggest that miR160 may also mediate these hormone signal interactions.

### Expression of eTMs, miR160, and *ARF10*, *-16*, and *-17* in ‘Sijimi’ Tissues

**FIGURE 11:**
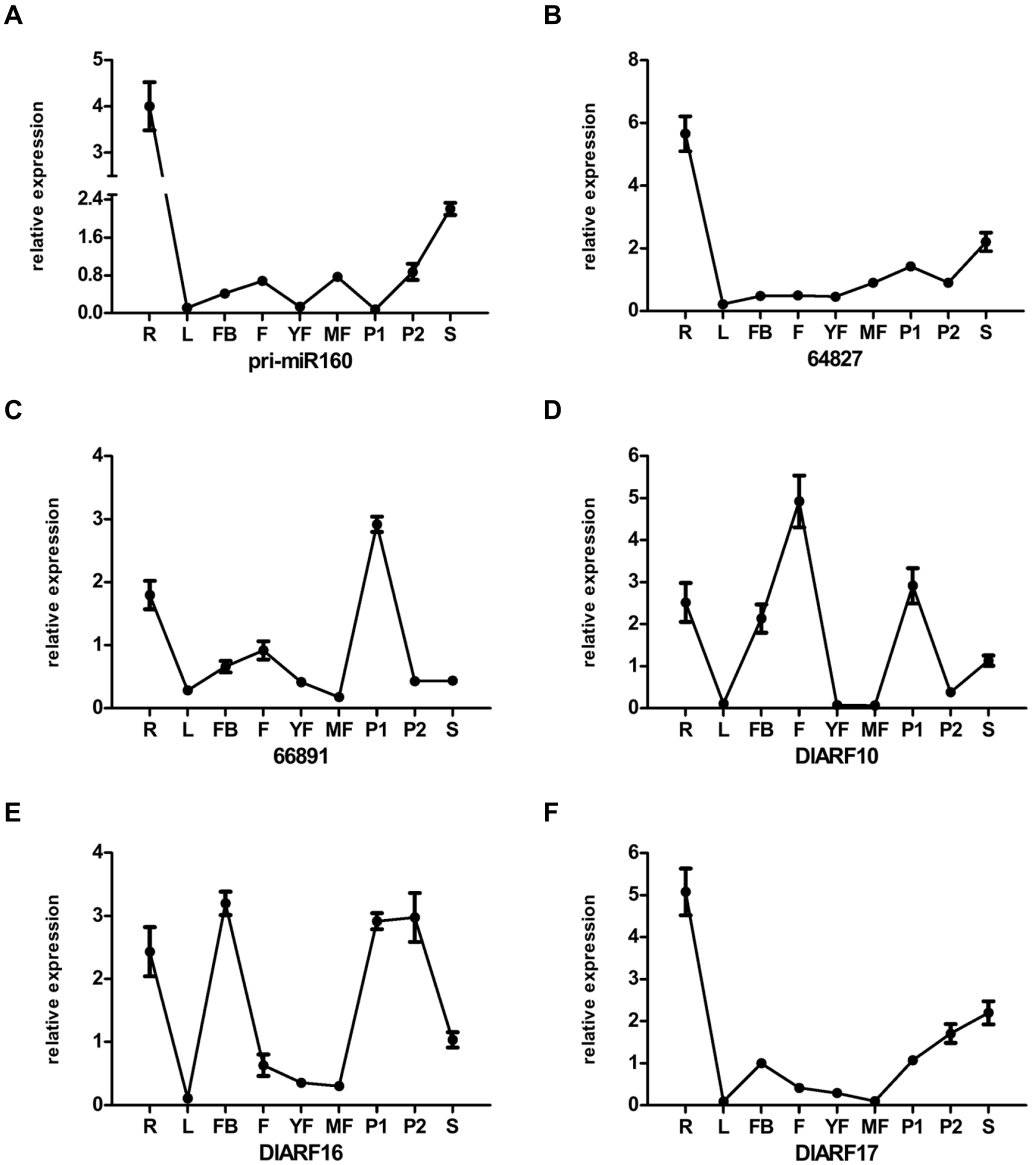
**Differential expression analysis of eTMs, miR160, and *DlARF10*, *-16*, and *-17* in vegetative and generative tissues of ‘Sijimi’ longan. (A–F)**, qPCR analysis of the expression levels of the eTMs, miR160, and *DlARF10/-16/-17*. Unigene64827: glucan endo-1,3-beta-glucosidase-like protein 2, Unigene66891: calpain-type cysteine protease DEK1 family. The expression levels were normalized to the reference genes *DlFSD1a*, *EF-1a*, and *eIF-4a*. Samples: roots (R), leaves (L), floral buds (FB), flowers (F); young fruit (YF), mature fruit (R), pericarp (P1), pulp (P2), and seeds (S).

**FIGURE 12:**
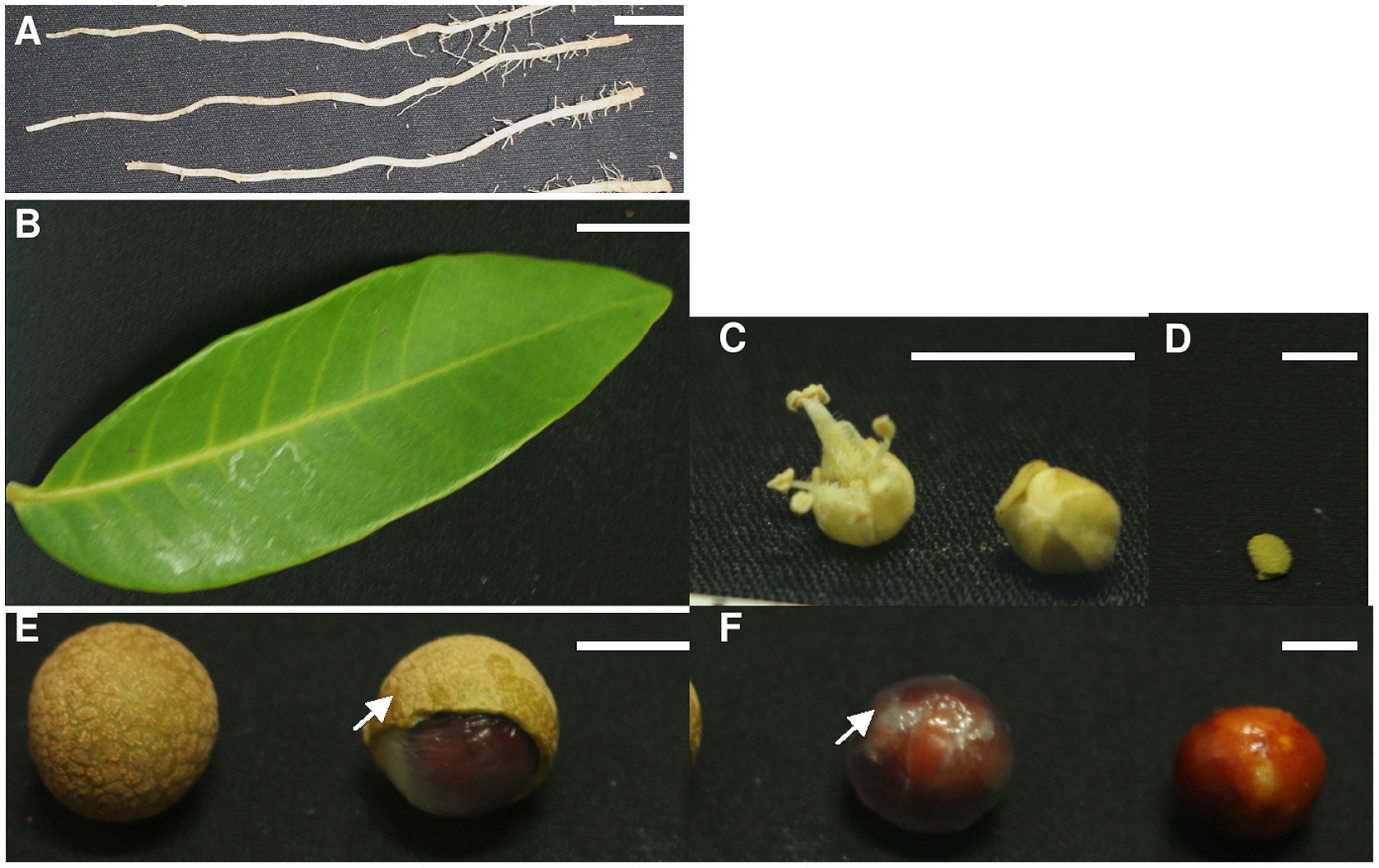
**Morphology of vegetative and generative tissues of ‘Sijimi’ longan. (A)** Roots (R); **(B)** leaves (L); **(C)** flowers (F) and floral buds (FB); **(D)**, young fruit (YF); **(E)** mature fruit (R) and pericarp (P1, arrow); **(F)** pulp (P2, arrow), and seeds (S). Bars are 8 mm.

To get more information about the characteristics of the eTM, miR160, and *ARF10*, -*16*, and *-17* accumulation patterns in other tissues, their expression in *D. longan* Lour. cv. Sijimi was studied (**Figures 11** and **12**). ‘Sijimi’ is unique cultivar of longan that blossoms and bears fruit throughout the year. A variety of tissues (**Figure 12**), including roots (R), mature leaves (L), floral buds (FB), flowers (F), young fruits (YF), mature fruits (MF), pericarp (P1), pulp (P2) and seeds (S), were used to assay RNA accumulation.

We examined the expression patterns in all tissues and found that the eTMs, miR160 and *ARF10*, *-16*, and *-17* exhibited tissue specificity (**Figures 11A–F**). All the genes were highly expressed in roots, and showed the lowest expression levels in mature leaves, except for calpain-type cysteine protease DEK1 (Unigene 66891), which was least expressed in mature fruits. These observations indicated that eTM-miR160-*ARF10- 16-17* signal transduction might affect ‘Sijimi’ root development. In addition, high expression levels were observed for pri-miR160, endo-1,3-beta-glucosidase-like protein 2-like (Unigene 64827) and *DlARF17* in seeds, calpain-type cysteine protease DEK1 (Unigene 66891) and *DlARF10* and *-16* in pulp, *DlARF10* in flowers, and *DlARF16* in floral buds and pericarp. Furthermore, the expression of pri-miR160 in young fruits and pulp, *DlARF10* and *-16* in young fruits and mature fruits, and *DlARF17* in mature fruits was very low or undetectable. These data suggested eTM-miR160-*ARF10-16-17* signal transduction plays an extensive role in ‘Sijimi’ development, but there was no significant negative correlation among the levels of eTM, miR160 and *DlARF10*, *-16*, and *-17* mRNA accumulation in the organs analyzed.

## Discussion

### Unique Size and Structure of longan miR160

Plant miRNAs are usually encoded by small gene families of up to 14 members (Parizotto et al., 2004), similar to protein-coding genes. The miR160 family in plants includes three members in *Arabidopsis*, six members in *O. sativa* and *Zea mays*, and seven members in *Populus trichocarpa* (Jin et al., 2012). In our study, only two members, dlo-miR160a and dlo-miR160d^∗^, were detected in longan somatic embryos. However, these two members were represented by 12 variants with identical or nearly identical sequences and different read counts. qPCR results further indicated that the different members of the dlo-miR160 family had different expression patterns. A previous study also showed that the multi-copy miR160 family has expression diversification, and may have functional diversity. For example, miR160a regulates embryo development at the very beginning of embryogenesis (Liu et al., 2010b), while miR160c regulates root cap development (Wang et al., 2005), which suggests that the different spatial and temporal expression patterns of the dlo-miR160 family members reflects their functional diversity in longan SE.

The secondary structures of miRNA genes play an integral role in their maturation, regulation and functions (Parizotto et al., 2004; Jiang et al., 2006). miRNA genes have been identified using computational approaches that depend upon the availability of reference or genomic sequences (Megraw et al., 2006; Meng et al., 2009; Branscheid et al., 2011; Han et al., 2014). However, most miRNA genes, especially in non-model plants, have not been investigated, primarily because of a lack of genome-wide datasets. In our study, 5′ and 3′ RACE experiments, together with the data deposited in the GenBank and miRBase databases, were used to obtain the full-length pri-miRNA for miR160. The corresponding pri-miR160a genomic DNA sequence (424 bp, containing one intron) was obtained, which was shorter than the *Arabidopsis* miR160a gene (2034-bp long; one intron), but longer than the *Arabidopsis* miR160b gene (378-bp long; no introns) (Szarzynska et al., 2009). This suggests that the pri-miR160 genes from different species and different members vary in size and structure.

### The Longan miR160 Promoter is Associated with Hormone Signal Transduction

Many previous studies have focused on elucidating the mechanism of miRNA target gene regulation, but there is limited information on the regulation of miRNA genes themselves. Previous studies have shown that the promoters of most of the known pre-miRNAs share similar characteristics with RNA polymerase II-dependent protein-coding gene promoters (Zhou et al., 2007). The identification of the promoters of miRNA genes is a work in progress; however, most studies have concentrated on *A. thaliana* (Megraw et al., 2006; Zhou et al., 2007) and *O. sativa* (Cui et al., 2009; Meng et al., 2009). Here, the pri-miR160 promoter was cloned from longan EC and contained the conserved TATA and CAAT boxes.

The miR160 family has the highest number of identified ARF binding sites (AuxREs) in cassava (Perez-Quintero et al., 2012), *G. max* (miR160d and miR160f) (Han et al., 2014), and rice (osamiR160f). However, ARF binding sites were not found in the longan miR160 promoter. Previous experiments showed that indole acetic acid (IAA) treatment did not influence the auxin-related rice homologous miRNAs significantly, such as osa-miR160, osa-miR164, and osa-miR167 (Meng et al., 2009). Here, exogenous 2,4-D treatment significantly influenced pri-miR160 expression, suggesting that ARF elements may be present outside of the 960 bp promoter region. Indeed, AuxREs in the 3′ regulatory region of miR160a are required for auxin regulation of miR160a expression in *Arabidopsis* (Liu et al., 2010b). However, AuxREs were not found in the 3′ regulatory region of pri-miR160.

Other *cis*-regulatory elements associated with plant hormones, such as ABA (ABRE) and SA (TCA-element), necessary for plant responses to various abiotic stresses (Kruszka et al., 2012), were detected in longan. In *Arabidopsis*, miR160, miR393 and miR397b were up-regulated, whereas miR169 and miR398 were down-regulated, in response to ABA stress. In another study, negative regulation of *ARF10* by miR160 modulated the response to ABA stress during germination (Liu et al., 2007). Our qPCR results showed that ABA up-regulated the expression of miR160, which suggests that miR160 is involved in ABA signaling in longan. Furthermore, pri-miR160 was down-regulated in response to SA treatment, implying that miR160 is also involved in SA signaling in longan. GA_3_, ET, and MeJA also up-regulated the expression level of pri-miR160, but corresponding *cis*-regulatory elements were not identified in the 960 bp longan miR160 promoter region. However, gibberellin response factor elements were found in the putative promoter regions of miR160f, miR160g, and miR160h in *G. max* (Han et al., 2014), and auxin-, MeJA-, ET-, GA_3_-, and SA-responsive elements were found in the promoter regions of miR160s in cassava (Pinweha et al., 2015). Taken together, these results clearly demonstrated that the pri-miR160 was down-regulated in response to SA (75 μM), but up-regulated by MeJA (50 μM), ET (30 ppm), GA_3_ (35 μM), or ABA (100 μM), suggesting that miR160s are widely involved in hormone signal transduction in longan. However, it is not clear whether these hormones affect the morphogenesis of somatic embryos; this requires further experimental validation.

The other types of *cis*-regulatory elements in the upstream region of the miR160 gene in longan were defense and stress responsive, such as the anaerobic-response element (ARE) and HSE, which were also found in the cassava miR160 promoter (Pinweha et al., 2015). Recently, reports showed that miR160 plays a role in the defense and stress responses of plants (Jin et al., 2012; Pinweha et al., 2015). miR160s were highly induced after infection with *Pseudomonas syringae* pv. tomato (Fahlgren et al., 2007). These results suggest that miR160 might play a role in defense and stress in plants.

In conclusion, these results form the basis for further experiments to identify the function of miR160 in responses to various hormone and environmental stimuli.

### eTMs Down-regulated miR160 Cleavage of *DlARF10*, *-16*, and *-17* mRNAs During Longan SE

Target mimicry, a new regulatory mechanism for miRNA functions, was first studied in *Arabidopsis*. eTMs for miR160 have been identified in *Arabidopsis* (Wu et al., 2013), rice (Wu et al., 2013), and *P. trichocarpa* (Shuai et al., 2014), and are functional target mimics that cause smaller and serrated leaves in transgenic plants (Wu et al., 2013). However, previous studies focused mainly on eTMs derived from intergenic non-coding RNA genes. In our study, four potential eTMs derived from annotated genes were initially identified for miR160a^∗^ in longan; however, only two eTMs: glucan endo-1,3-beta-glucosidase-like protein 2-like (Unigene64827) and calpain-type cysteine protease DEK1 (Unigene 66891), were detected during longan SE by qPCR. Their expression patterns were inversely proportional to that of the corresponding dlo-miR160a^∗^, especially at the GE to CE stages. Moreover, their expression was down-regulated while dlo-miR160a^∗^ was up-regulated by auxin and ABA treatment. These results further proved that Unigene64827 and Unigene 66891 inhibited the expression level of dlo-miR160a^∗^ and participated in auxin and ABA signal transduction during longan SE. Unigene64827 encodes a homolog of glucan endo-1,3-beta-glucosidase-like protein 2, which is related to cell wall synthesis (Meitzel et al., 2011). Unigene66891 encodes a protein similar to calpain-type cysteine protease DEK1, which is required for epidermal development in the embryo (Johnson et al., 2005). These results suggest that they play a potential role in cell wall synthesis and epidermal development in the embryo by regulating dlo-miR160a^∗^, especially at the GE to CE stages. This hypothesis requires further experimental validation.

miR160 and its target ARFs are conserved in dicots and monocots (Rhoades et al., 2002). Here, *DlARF10*, *-16*, and *-17* targeting by dlo-miR160a was confirmed, and their expression levels were mostly the reverse of that of dlo-miR160a. In addition, dlo-miR160a was up-regulated while *DlARF16* and *-17* were down-regulated by 2,4-D treatment. These results were consistent with earlier reports (Liu et al., 2010b), which suggested that the increased expression of dlo-miR160a induced by 2,4-D treatment down-regulated the expression of *ARF16* and *-17*, further confirming that miR160-ARF16-17 auxin signal transduction operates in longan similarly to *Arabidopsis*. By contrast, *DlARF10* was up-regulated after 2,4-D treatment, suggesting it may not be controlled by dlo-miR160a under auxin treatment.

Auxin response factor proteins can either activate or repress transcription, based on their protein structures and transient expression experiments. ARFs 5–8 and ARF19 contain Gln-Leu-Ser–rich middle regions that may function as transcriptional activators, whereas ARFs 1–4, 9–18, and 20–22 possibly act as repressors, with Pro-Ser-Thr–rich middle regions (Tiwari et al., 2003; Mallory et al., 2005). In our study, *DlARF10*, *-16*, and *-17* expression was stronger in whole embryos at the EC and ICpEC stages, and gradually decreased during somatic embryo development, suggesting they might function as repressors to establish and maintain auxin signals during embryogenesis (Mallory et al., 2005; Liu et al., 2007, 2010b).

miR160-resistant mutants have been used successfully to characterize the biological functions of *ARF10* (Liu et al., 2007), *-16* (Wang et al., 2005) and *-17* (Mallory et al., 2005), revealing that down-regulation of these genes is essential to maintain the normal developmental programs of the roots, leaves, flowers and siliques. In our study, neither pri-miR160 nor *ARF10*, *-16*, or *-17* exhibited tissue specificity in ‘Sijimi’ longan. They all showed high expression in roots and were not detected in mature leaves. By contrast, miR160c expression was low or undetectable in juvenile leaves and became high in older leaves, as assessed by GUS staining of ProMIR160c:GUS plants (Wang et al., 2005). miR160a is expressed in all organs, especially in reproductive organs (Liu et al., 2010b), suggesting that miR160 regulation of *ARF10*, *-16*, and *-17* signal transduction has an extensive role in ‘Sijimi’ development. However, there were no remarkable negative correlations among the levels of miR160 and the *DlARF10*, *-16*, and *-17* mRNAs in the analyzed tissues.

## Conclusion

**FIGURE 13:**
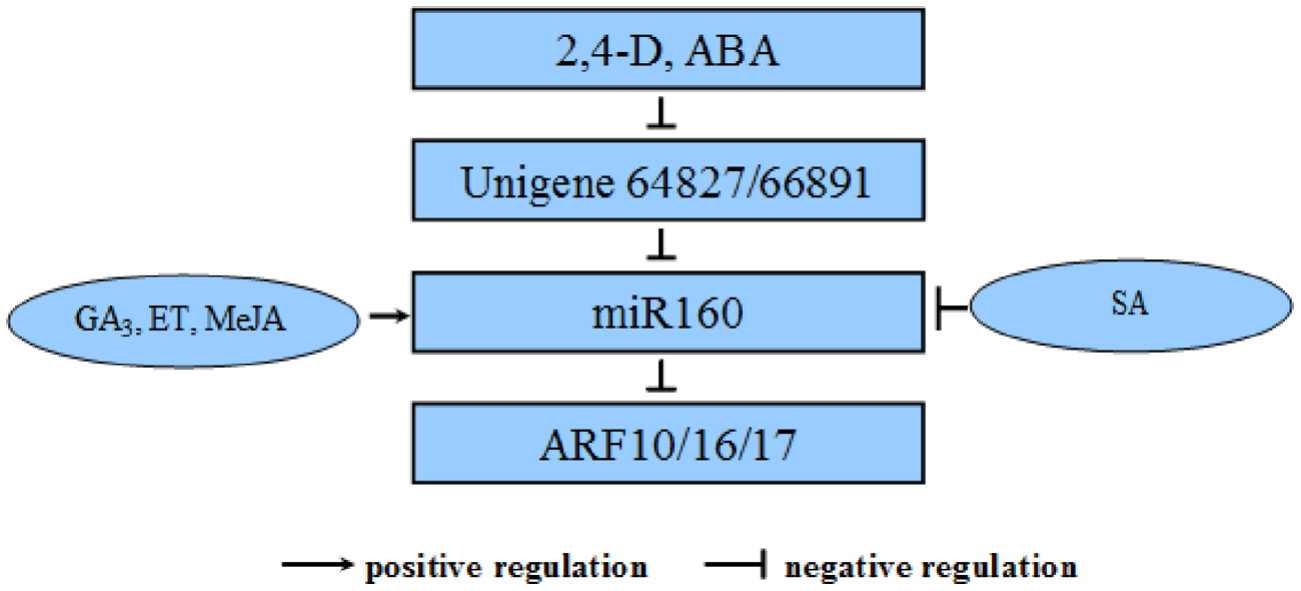
**Negative regulatory relationships among the eTMs, miR160, and its targets involved in SE in *D. longan* in relation to hormone metabolism.** The eTMs Unigene64827 and Unigene66891 belong to the glucan endo-1,3-beta-glucosidase- like protein 2 and calpain-type cysteine protease DEK1 families, respectively. They down-regulate miR160 by mediating cleavage of *ARF10*, *-16*, and *-17*, which are involved in SE in *D. longan*, and respond to 2,4-D and ABA signal transduction. In addition, the expression of pri-miR160 is controlled by exogenous GA_3_, ET, MeJA and SA.

In summary, we performed dlo-miR160 family member identification, primary transcript and promoter cloning; target mimic prediction, miRNA target identification, and qPCR verification. According to our experiments as well as the negative feedback model, we propose a possible “eTM–miR160–target” regulatory loop during longan SE (**Figure 13**). The eTMs glucan endo-1,3-beta-glucosidase-like protein 2-like (Unigene64827) and calpain-type cysteine protease DEK1 (Unigene 66891) down- regulation of miR160’s mediation of *ARF10*, *-16*, and *-17* cleavage is associated with SE, and responds to 2,4-D and ABA signaling transduction in longan. In addition, the expression of pri-miR160 is controlled by GA_3_, ET, MeJA, and SA.

## Author Contributions

YL conceived the study, participated in its design, carried out the experimental work and wrote the manuscript. ZL conceived the study, participated in its design and coordination, and helped to draft the manuscript. QT, LL, RL, MY, DZ, YC, and ZZ prepared the materials. All authors read and approved the final version of the manuscript. All authors agree to be accountable for all aspects of the work in ensuring that questions related to the accuracy or integrity of any part of the work are appropriately investigated and resolved.

## Conflict of Interest Statement

The authors declare that the research was conducted in the absence of any commercial or financial relationships that could be construed as a potential conflict of interest.

## Abbreviations

2,4-D: 2,4-dichlorophenoxyacetic acid
ABA: abscisic acid
ARF: auxin response factor
CE: cotyledonary embryos
EC: friable embryogenic callus
ET: ethylene
eTMs: endogenous target mimics
GA_3_: gibberellic acid
GE: globular embryos
HE: heart- shaped embryos
ICpEC: incomplete compact pro-embryogenic cultures
MeJA: methyl jasmonate
miRNAs: microRNAs
pre-miRNA: precursor miRNA
pri-miRNAs: primary miRNA transcripts
qPCR: real-time quantitative PCR
RLM-RACE: RNA ligase-mediated rapid amplification of cDNA ends
SA: salicylic acid
SE: somatic embryogenesis
TE: torpedo-shaped embryos
UTR: untranslated region
